# The Interleukin-17 Induced Activation and Increased Survival of Equine Neutrophils Is Insensitive to Glucocorticoids

**DOI:** 10.1371/journal.pone.0154755

**Published:** 2016-05-03

**Authors:** Ruby Yoana Murcia, Amandine Vargas, Jean-Pierre Lavoie

**Affiliations:** Department of Clinical Sciences, Faculty of Veterinary Medicine, Université de Montréal, Saint-Hyacinthe, Quebec, Canada; University Tuebingen, GERMANY

## Abstract

**Background:**

Glucocorticoids (GCs) are the most effective drugs for the treatment of human asthma. However, a subgroup of asthmatic patients with neutrophilic airway inflammation is insensitive to GCs. Interleukin-17 (IL-17), a cytokine upregulated in the airways of a subset of human asthmatic patients, contributes to the recruitment of neutrophils and induces a glucocorticoid resistance in human airway epithelial cells. We hypothesized that IL-17 similarly activates neutrophils and contributes to their persistence in the asthmatic airways in spite of glucocorticoid therapy.

**Objective:**

To determine whether IL-17 directly activates neutrophils and whether this response is attenuated by GCs.

**Methods:**

Neutrophils were isolated from the blood of horses and incubated in the presence of recombinant equine IL-17, LPS and dexamethasone. mRNA and protein expression of IL-17 receptors (IL-17RA/IL-17RC) were assessed by qPCR and immunoblot, respectively. Pro-inflammatory cytokine expression, cell viability and apoptosis were determined by qPCR, Trypan Blue exclusion test, and flow cytometry, respectively.

**Results:**

Equine neutrophils express both IL-17RA and IL-17RC at the mRNA and protein levels. Neutrophil stimulation with IL-17 increases the mRNA expression of IL-8, which is not attenuated by dexamethasone (p = 0.409). Also, neutrophil viability is significantly increased (p<0.0001) by IL-17 in the presence of LPS when compared to LPS alone. Flow cytometry and light microscopy revealed that LPS-induced apoptosis is decreased by IL-17 (p = 0.02 and p = 0.006 respectively).

**Conclusion:**

These results indicate that IL-17 directly activates equine neutrophils at 24 hours, and that the expression of IL-8 thus induced is not attenuated by GCs. Additionally, IL-17 increases neutrophil viability and decreases apoptosis. These findings suggest an important role of IL-17 in pulmonary persistence of neutrophils in the asthmatic airways.

## Introduction

Glucocorticoids (GCs) are the most effective drugs for the treatment of many inflammatory and immune human diseases such as asthma, rheumatoid arthritis, and autoimmune diseases [1]. Their effects are induced primarily through a reduction in the expression of cytokines, chemokines, and adhesion molecules implicated in the recruitment and activation of inflammatory cells to the site of inflammation [2]. Cohorts of patients with neutrophilic asthma have been linked to a relative resistance to the action of GCs [3]. The persistence of neutrophils in the bronchial lumen could maintain the inflammatory process that leads to mucus hypersecretion and airway remodeling [4]. Upon activation by various mediators, neutrophils synthesize proteins involved in their immunological response, including a variety of chemokines, that are potentially implicated in the recruitment of distinct leukocyte subpopulations [5]. Interleukin-17 (IL-17) is produced by CD4 + T helper 17 and other cell types such as gamma delta T cells (γδ T), natural killer cells (NK), lymphoid tissue inducer cells (LTi), macrophages, eosinophils, and neutrophils [6, 7]. IL-17 is capable of promoting indirectly the activation and recruitment of neutrophils into the airways by inducing the production of chemokines such as IL-8, CXCL1, and G-CSF in endothelial and epithelial cells [8]. It has been demonstrated that IL-17 signals through a heterodimeric complex receptor composed of IL-17RA and IL-17RC receptor [9, 10]. Studies reported that the IL-17RA subunit of the IL-17 receptor is constitutively present in human neutrophils, and that these cells may express IL-17RC when stimulated [11]. As IL-17 is upregulated in the airways of human asthmatics, and stimulation of human bronchial epithelial and mononuclear cells with IL-17 resulted in hypo-responsiveness to GCs [12, 13], we hypothesized that IL-17 contributes to the insensitivity of neutrophils to GCs in asthmatics airways. This study aimed to evaluate the contribution of IL-17 to neutrophil proinflammatory gene expression, their response to GCs, and whether IL-17 modulates neutrophil viability and apoptosis. We studied equine neutrophils because the cytokine response and neutrophil biology in this species has marked similarities with human, and the large amount of cells required for these studies can easily be obtained from the same animal. Indeed, horses spontaneously develop heaves, a neutrophilic asthma-like disease [14] associated with an upregulation of IL-17 [15], and the airway neutrophilia is unaffected by the administration of GCs [16].

## Materials and Methods

### Ethics Statement

Eight mixed breed healthy mares were studied (mean age 11.1 years (range 6–20); mean weight 512 kg (range 490–550). Mares were part of the research herd at the Faculty of Veterinary Medicine of the Université de Montréal. All animal experimental procedures were performed in accordance with the guidelines of the Canadian Council on Animal Care and were approved by the Animal Care Committee of the Faculty of Veterinary Medicine of the Université de Montréal (Rech-1716).

### Neutrophil and Neutrophil-depleted Leukocyte Isolation

Venous blood was collected from a jugular vein. Blood was placed into sterile heparinized blood collection tubes (Tyco healthcare, Pointe-Claire, QC, CA). The plasma rich layer was recovered after 30 minutes of sedimentation using a density gradient method of separation Ficoll-Paque^TM^ Premium 1084 (Fisher Scientific, Ottawa, ON, CA).

The leucocyte polymorphonuclear-depleted and polymorphonuclear-rich cell layers were both harvested, and the remaining erythrocytes were lysed by hypotonic treatment with ultrapure distilled water (Life technologies, Burlington, ON, CA). Cells were washed and suspended in a buffer solution containing PBS 1X, EDTA 0.5mM (Life technologies, Burlington, ON, CA), and BSA 0.2% (Sigma-Aldrich, St Louis, MO, USA). Counting and viability were achieved with the Trypan blue 0.4% exclusion method (Life technologies, Burlington, ON, CA) in a hemocytometer chamber. Purity was evaluated in cytospin stained slides for differential counting as previously reported [17].

Thereafter, positive immunomagnetic selection was performed as described previously [17]. Briefly, neutrophils were obtained from the polymorphonuclear-rich suspension by incubation with the monoclonal mouse anti-CD90 DH24A Monoclonal (VMRD, Pullman, WA, USA) and a secondary anti-mouse IgM antibody conjugated to paramagnetic microbeads MACS® (Miltenyi Biotec, Bergisch Gladbach, GER) at 25 μl/10^6^ cells. Suspension was passed through a LS column (Miltenyi Biotec, Bergisch Gladbach, GER) and the positive fraction (neutrophils) was recovered with a mean purity of 99.5% and viability of 99% for proinflammatory cytokine studies. Purity of 100% and viability of 99% were obtained for protein evaluation and genic expression of the IL-17 receptors.

### Cell culture

Freshly isolated peripheral blood neutrophils and polymorphonuclear-depleted cells were suspended at 5x10^6^/ml and cultured in 12- or 24-well plates (Corning Incorporated, Corning NY, USA) in RPMI medium supplemented with L-glutamine 200 mM, 100 U/mL penicillin, 100 U/mL streptomycin, and 10% of low endotoxin, heat-inactivated fetal bovine serum (Life Technologies, Burlington, ON, CA). Cells were incubated at 37°C in 5% CO_2_ in the presence or absence of recombinant equine IL-17 (reIL-17) (Cederlane, Burlington, ON, CA) at 1, 10 and/or 100 ng/ml, and in the presence or absence of LPS at 100 ng/ml (Sigma-Aldrich, Saint Louis, MO, USA) and incubated for 5h (subunits receptor and IL-17 direct activation studies).

For expression of pro-inflammatory cytokine at 5h (n = 5) and 24h (n = 6), cells were cultured in presence or absence of dexamethasone (DEX) at 10^-6^M (Sigma-Aldrich), LPS and/or reIL-17 at 100 ng/ml. Recovered cells (neutrophils and mononuclear cells) were lysed in Isol-RNA lysis reagent (Fisher Scientific, Ottawa, ON, CA) or RIPA buffer (Sigma Aldrich) for mRNA or proteins analysis, respectively.

### Gene expression study

mRNA expression of the two subunits of the IL-17 receptor (IL-17RA and IL-17RC) and of selected pro-inflammatory cytokines (IL-6, IL-8, TNF-α and IL-1β) were measured at 5 and 24 hours by qPCR. mRNA was extracted from Isol-RNA according to manufacturer’s instructions (Fisher Scientific, Ottawa, ON, CA). Purity and concentration were assessed using a spectrophotometer Nanodrop ND1000 (Fisher Scientific, Waltham, MA, USA). One microgram of total mRNA was reverse transcribed as described previously [18].

Quantitative PCR (qPCR) reactions were performed using QuantiTect SYBR Green PCR Kit (Qiagen, Toronto, ON, CA) according to the manufacturer's instructions with the Rotor-Gene RG3000 (Corbett Research, Sydney, AS) as described before [19]. Briefly, 1 μl of cDNA was used in 20 μl of final volume PCR reaction, containing 0.5 μM each of sense and antisense primers and 2.75 mM MgCl_2._ Primers were designed to span exon–intron boundaries to prevent amplification of genomic DNA (Table 1). Samples were run in duplicate with an appropriate negative control.

**Table 1:** Sequences of primers used for qPCR assays.

Gene Id	Forward sequence (5’-3’)	Reverse sequence (5’-3’)
**IL-17RA**	ACTCAAGCACACACCAGAGG	TGTGTCTGAGGCAGTCGTTC
**IL-17RC**	TGGCCCTTGAATTCCCATTGCT	CTGGGTTCCAAGGCACAGAATGAT
**IL-1β**	AGACAACAGTGAAGTGCAGCCT	GACTGACAAGATACCTGTGGCCT
**IL-6**	TCACTCCAGTTGCCTTCTCC	CCAGATTGGAAGCATCCGTC
**IL-8**	GCAGACCTCAGCTCCGTTGAC	CTTTCTGCAGCTCTGTGTGAAG
**TNF-α**	ATCCGAGATGTGGAGCTGGC	GACTGGAAGGCATTCGGTAACT

### Immunoblot analysis

Neutrophils and mononuclear cells were lysed in 150μl RIPA buffer, according to the manufacturer’s instructions. Protease and phosphatase inhibitor cocktails were added (Life technologies, Burlington, ON, CA) and samples were sonicated (Cole Palmer Ultrasonic Processor, CPX 750). Protein quantification was achieved using the Pierce^TM^ BCA Protein Assay Kit (Life technologies, Burlington, ON, CA). Protein suspensions were frozen at -80°C for further protein assays. Equal amounts of protein (30 μg per lane) were separated by size on 12% Mini-PROTEAN® TGX™ Precast Gels (Bio-Rad Laboratories, Mississauga, ON, CA) and transferred to a solid PVDF membrane (Fisher Scientific, Ottawa, ON, CA). Immunodetection was performed using anti-IL-17RA ARP47009_P050 (Cederlane, Burlington, ON, CA) and anti-IL-17RC (L-12): sc-99936 (Santa Cruz Biotechnology, Dallas, TX, USA). Anti-rabbit IgG HRP-Linked antibody was used as a secondary antibody (New England Biolabs, Whitby, ON, CA). Signal was enhanced by SuperSignal West Dura (Fisher Scientific, Ottawa, ON, CA). Protein bands were scanned using a chemiluminescence with a Fusion Fx Vilber Lourmat (Montreal Biotech Inc., Dorval, QC, CA).

### Flow cytometry and morphological evaluation of apoptosis

APC Annexin V staining was performed as described previously [17]. Briefly, one hundred microliters of the cell suspension were incubated with 5 μl of APC Annexin V, an apoptosis cell marker, and 5 μl of 7-Aminoactinomycin D (7-AAD), a necrosis cell marker (BD Pharmingen, San Diego, CA, USA). Cell apoptosis levels were analyzed using CellQuest Pro software on a FACScalibur instrument (BD Biosciences, Mississauga, ON, CA). Data were collected from 10,000 events gated on granulocytes. Analyzed unstained cells as well as single marker-stained cells were used to set photomultiplier voltage and compensation parameters for fluorescence detection in FL-3 and FL-4 channels. APC Annexin V-negative and 7-AAD-negative cells were considered viable.

Morphologic evaluation of apoptosis was performed on cytopreparations (5x10^4^ cells) stained with Protocol Hema 3 (Fisher Scientific, Ottawa, ON, CA) by one blinded investigator. The percentage of apoptotic cells was evaluated on 500 cells by light microscopy [20].

### Statistical analysis

Repeated measures ANOVA and *a priori* comparison with Fisher method was used to evaluate cytokine mRNA expression and apoptosis of neutrophils. For the detection of IL-17 receptor subunits in neutrophils and mononuclear cells, data were analyzed with paired student t-tests. Results were expressed as the mean ± S.D. The software GraphPad Prism 6 was used for the analysis and p<0.05 was considered significant.

## Results

### Neutrophils express both subunits of IL-17 receptor

**Fig 1:**
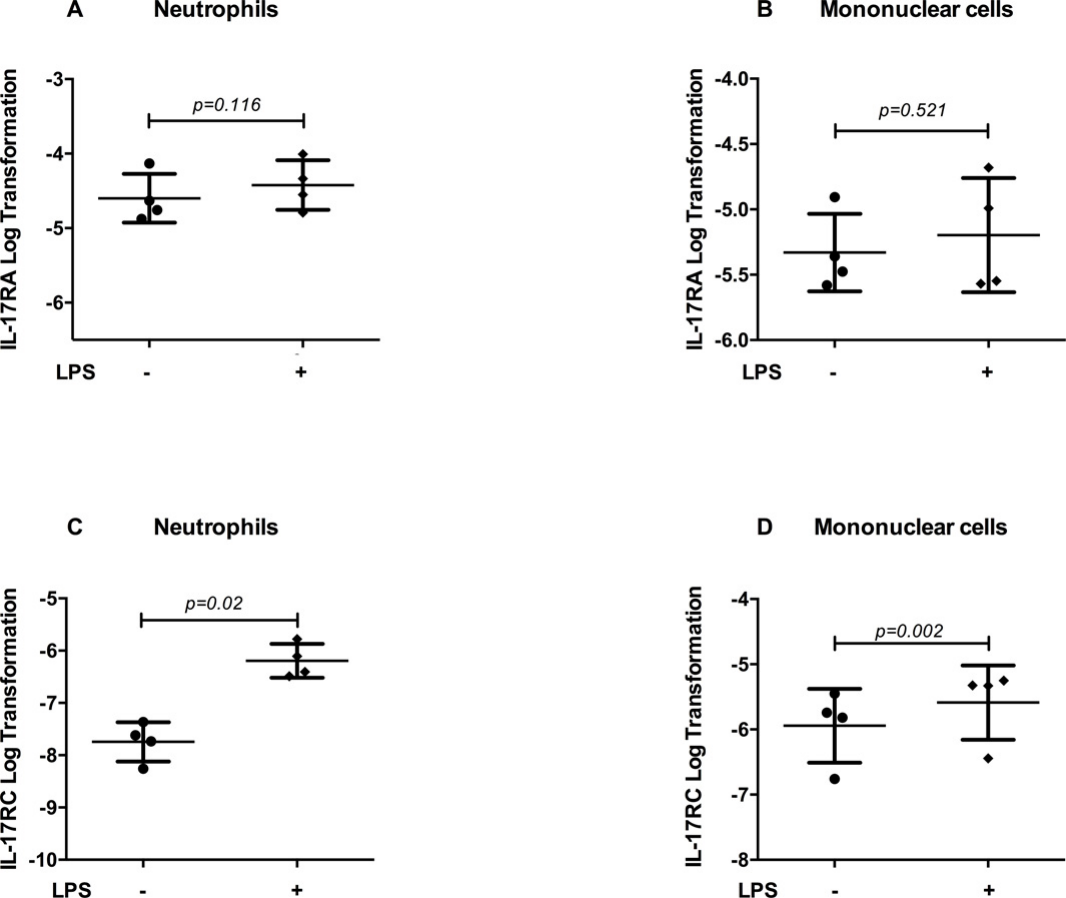
mRNA expression of IL-17 receptor subunits in neutrophils and mononuclear cells. Equine neutrophils (n = 4) and mononuclear cells (n = 4) were isolated from peripheral blood and cultured (5 hours) in presence or absence of LPS. IL-17RA (A and B) and IL-17RC (C and D) mRNA expression were evaluated by qPCR. Each symbol represents the mean of an experiment run in duplicate for every horse.

**Fig 2:**
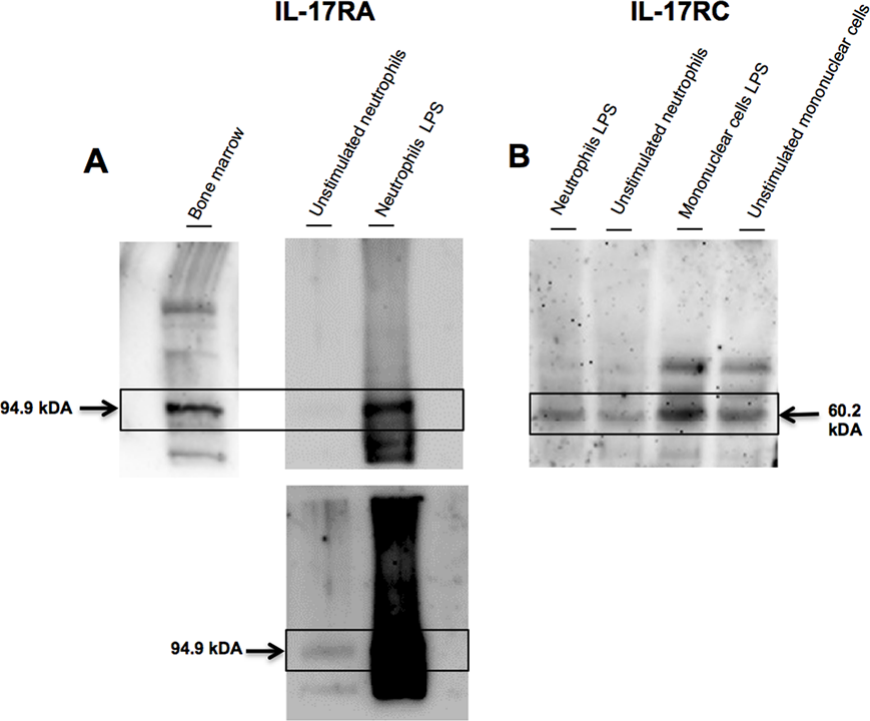
Protein expression of IL-17 receptor subunits in neutrophils. Equine neutrophils (n = 1) were isolated from peripheral blood and cultured (5 hours) in presence or absence of LPS. (A). IL-17RA and (B). IL-17RC proteins were detected by immunoblot. Mononuclear cells (IL-17RC) and bone marrow (IL-17RA) were used as positive controls. **Lower panel.** The membrane was overexposed to improve the visibility of IL-17RA in unstimulated neutrophils.

Equine neutrophils expressed IL-17RA and IL-17RC at mRNA (Fig 1A, n = 4) and protein levels (Fig 2A, n = 1), in both the presence or absence of LPS (5 hours). LPS-stimulation significantly increased IL-17RC mRNA expression in both neutrophils (p = 0.02, Fig 1C) and mononuclear cells (p = 0.002, Fig 1D), while the expression of IL-17RA remained unchanged (neutrophils, p = 0.116, Fig 1A; mononuclear cells, p = 0.521, Fig 1B).

### The reIL-17 does not activate neutrophils and dexamethasone does not attenuate the response of IL-8 and IL-6 induced by LPS after 5 hours

**Fig 3:**
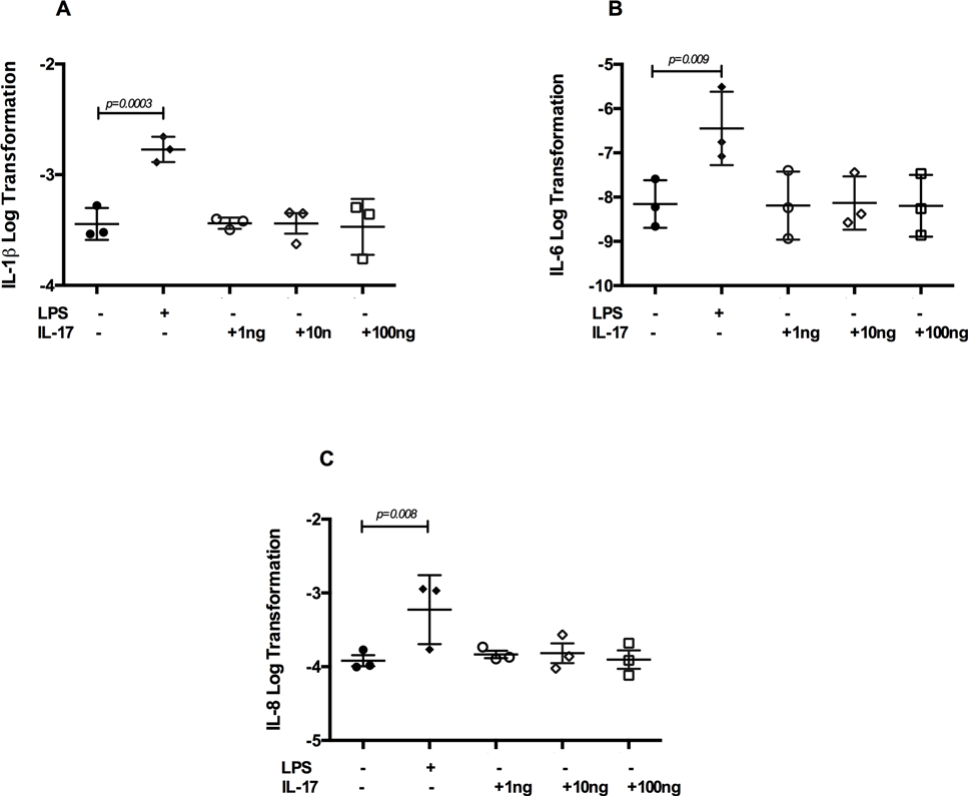
IL-17 does not directly activate neutrophils at 5 hours. Equine neutrophils (n = 3) were isolated from blood and cultured (5 hours) in presence or absence of IL-17 at different concentrations (ng.ml^-1^). (A). IL-1β, (B). IL-6 and (C) IL-8 mRNA expression were evaluated by qPCR. Upregulation of all cytokines was observed in LPS-stimulated but not in IL-17-pretreated neutrophils. Each symbol represents the mean of an experiment run in duplicate for every horse.

**Fig 4:**
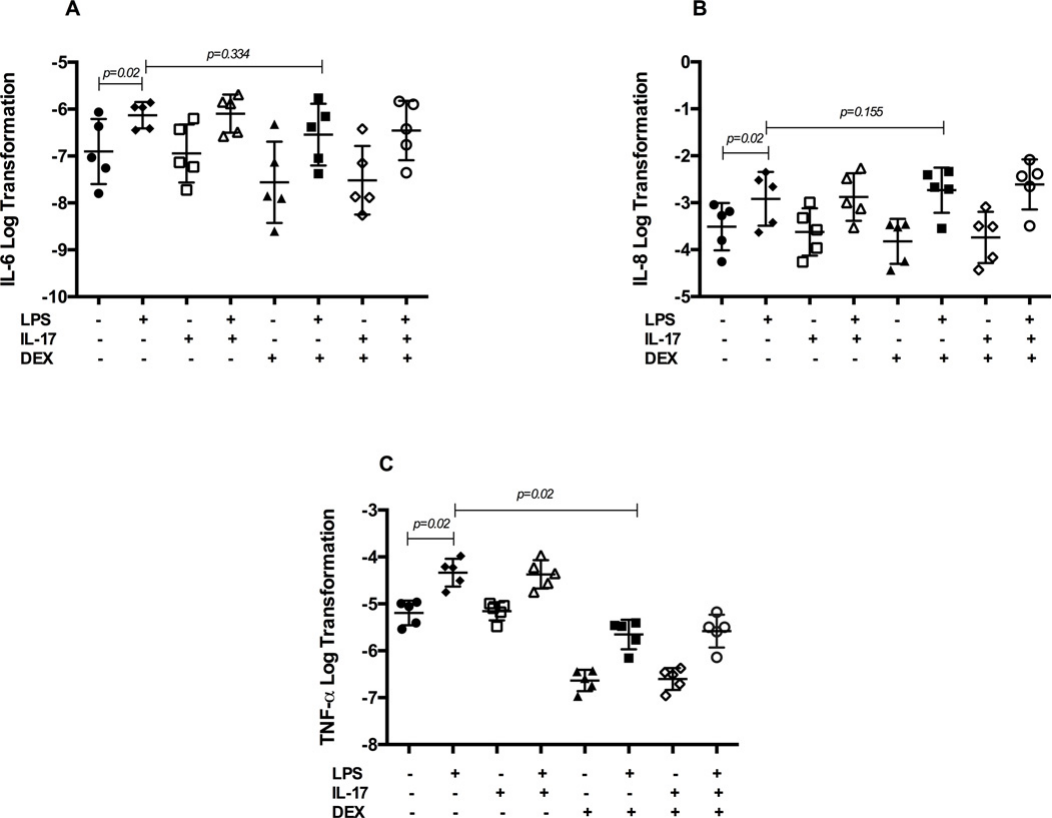
Pro-inflammatory cytokines expression in neutrophils at 5h. Equine neutrophils (n = 5) were cultured (5 hours) in presence or absence of IL-17, LPS and dexamethasone (DEX). (A). IL-6 (B). IL-8 and (C). TNF-α mRNA expression were evaluated by qPCR. Each symbol represents the mean of an experiment run in duplicate for every horse.

LPS induced a significant increase in the mRNA expression of IL-1β, IL-6, IL-8 and TNF-α after 5 hours of stimulation compared to unstimulated neutrophils (Figs 3 and 4). Expression of these cytokines was unaffected by a pretreatment with reIL-17. Expression of IL-6 and IL-8 was not attenuated by DEX (p = 0.334 and 0.155 respectively) (Fig 4A and 4B). DEX down-regulate LPS-induced TNF-α (p = 0.02) mRNA expression (Fig 4C).

### Neutrophil activation is induced by reIL-17 which is not inhibited by glucocorticoids at 24 hours

**Fig 5:**
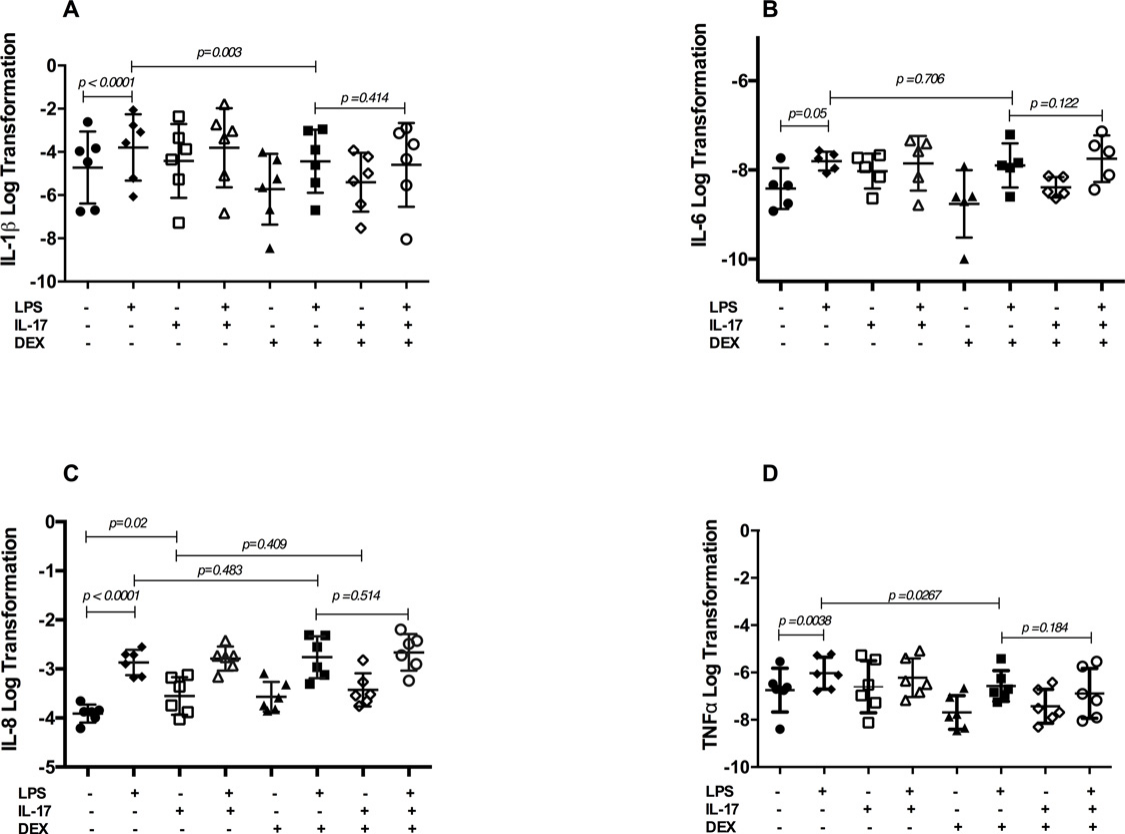
Pro-inflammatory cytokines expression in neutrophils at 24 hours. Equine neutrophils (n = 6) were cultured (24 hours) in presence or absence of IL-17, LPS and dexamethasone (DEX). (A). IL-1β, (B). IL-6, (C). IL-8, and (D). TNF-α mRNA expression were evaluated by qPCR. Each symbol represents the mean of an experiment run in duplicate for every horse.

Expression of IL-8 mRNA was significantly upregulated by reIL-17 (Fig 5C, p = 0.02). This IL-8 mRNA expression was not attenuated by DEX (p = 0.409) (Fig 5C). LPS also induced a significant increase in mRNA expression of IL-1β, IL-6, and TNF-α after 24 hours of stimulation when compared to unstimulated neutrophils (Fig 5). Expression of these cytokines was unaffected by a pretreatment with reIL-17 at 24 hours. The LPS-induced IL-1β and TNF-α expression was downregulated by DEX (p = 0.003 and p = 0.027 respectively) (Fig 5A and 5D), but not the IL-6 and IL-8 mRNA expression (p = 0.704 and p = 0.483 respectively) (Fig 5B and 5C).

### The reIL-17 increases viability and decrease apoptosis of neutrophils

**Fig 6:**
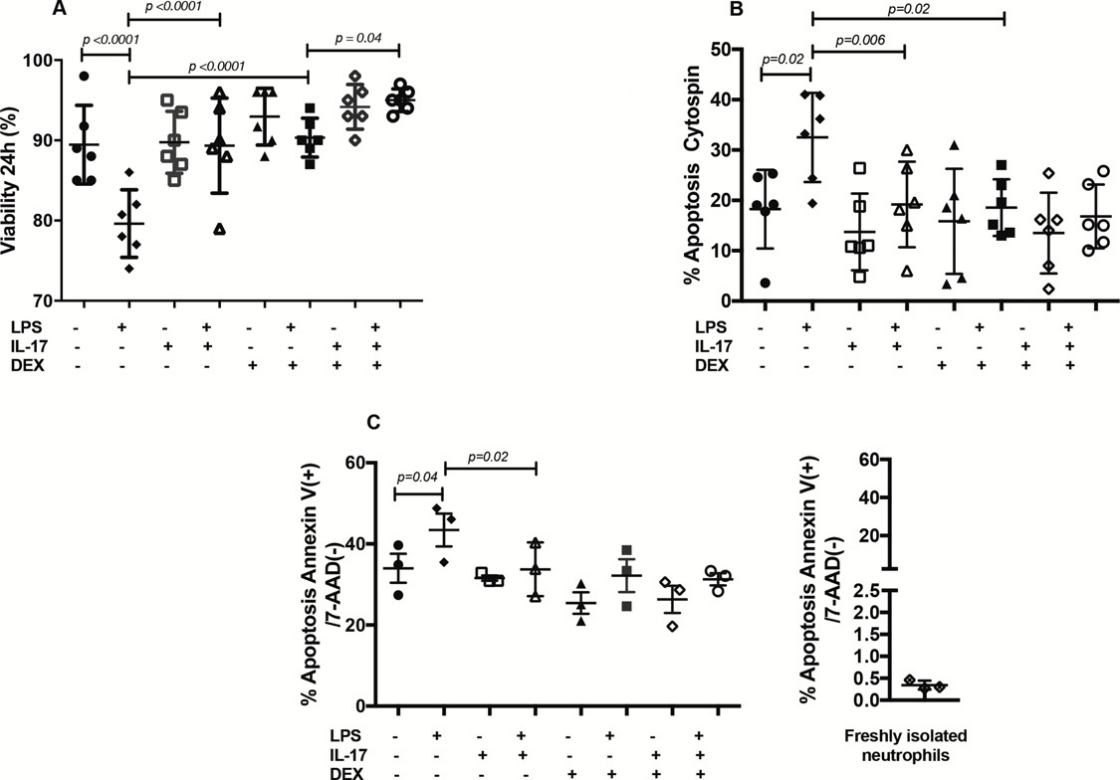
Assessment of neutrophil viability and apoptosis. Equine neutrophils were cultured (24 hours) in presence or absence of LPS, IL-17 and DEX. (A). Viability (n = 6) was evaluated by the trypan blue method. (B). Percentage of apoptotic cells (n = 6) was evaluated blindly by morphological evaluation on cytopreparations. (C). Percentage of apoptosis (n = 3) was evaluated by flow cytometry (Annexin V(+)/7-AAD(-)). Each symbol represents the mean of an experiment run in duplicate for every horse.

**Fig 7:**
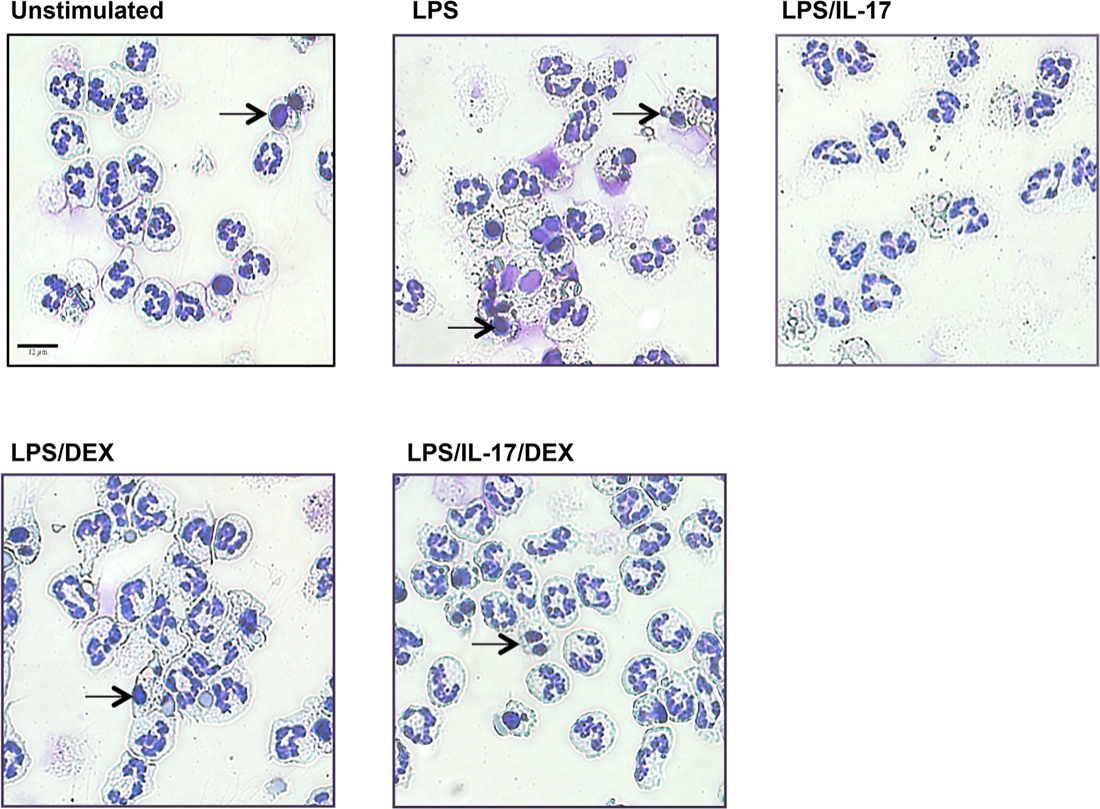
Morphological changes in cultured neutrophils (24h). Neutrophils were isolated and cultured 24 hours in presence or absence of LPS, dexamethasone (DEX) and IL-17. Morphological features of apoptosis were evaluated by a light microscope at 40X magnification (Protocol Hema 3 Stain). Arrows indicate apoptotic neutrophils, characterized by nuclear pyknosis and condensation of nuclear chromatin. Bar scale 12 *μ*m.

LPS induced decreased viability of neutrophils at 24 hours (Fig 6A), which was restored by both reIL-17 (p<0.0001) and DEX (p<0.0001). Importantly, adding reIL-17 further increased the DEX effect (p = 0.04) on the viability of LPS-stimulated neutrophils. Flow cytometry analysis (Fig 6C) and light microscopy (Figs 6B and 7) results indicated that both reIL-17 and DEX significantly decreased the apoptosis induced by LPS.

## Discussion

Previous studies have indicated that IL-17 is implicated in the indirect recruitment of neutrophils into the airways. In this study, we sought to determine whether IL-17 directly activates and induces a relative insensitivity of neutrophils to glucocorticoids by investigating its ability to regulate pro-inflammatory gene transcription, cell viability, and apoptosis. We first demonstrated the presence of both subunits of the IL-17 receptor in unstimulated, highly purified equine neutrophils. We then observed that reIL-17 directly activates the IL-8 mRNA expression of neutrophils at 24 hours, and that this activation is not attenuated by DEX. Furthermore, reIL-17 increased the viability of LPS-stimulated neutrophils and potentiated the dexamethasone-induced increase in viability of equine neutrophils. The significant inhibition of LPS-induced apoptosis in the presence of IL-17 suggests that an apoptosis delay mechanism appears to be involved. Taken together, these findings suggest a possible contribution of IL-17 to the persistence of neutrophils in the airways of glucocorticosteroids-treated patients.

### Insensitivity to glucocorticoids

Severe neutrophilic asthma is poorly responsive to GCs and neutrophils persist in the airways with this treatment [3, 21]. Considering that IL-17 expression is increased in sputum and bronchial biopsies [22, 23] of cohorts of asthmatics patients, and that this cytokine causes an insensitivity of bronchial epithelial cell to GCs [24], we sought to elucidate whether IL-17 exerts a similar role on neutrophils. We chose an equine model for our study due to the fact that horses are prone to developing a severe neutrophilic asthma-like condition associated with an upregulation of IL-17 within their airways [15, 25], in which the neutrophilic inflammation is poorly responsive to GCs [16, 26], as seen in some humans [27, 28].

In a response similar to that of human airway epithelial cells [12], IL-17-induced an upregulation of IL-8 mRNA, a potent neutrophil chemokine [29], in neutrophils stimulated for 24 hours. Interestingly, the IL-17-induced (24 hour) and LPS-induced (5 hour and 24 hour) IL-8 mRNA upregulation was not attenuated by dexamethasone. This finding suggests that IL-17 can contribute to continued neutrophil recruitment to the enflamed site and therefore to the persistence of neutrophils within the airways of GC treated patients. The neutrophil response to IL-17 may also contribute to asthmatic airway remodeling, as IL-8 has been shown to promote airway smooth muscle (ASM) proliferation by enhancing the number and survival of ASM cell *in vitro* [30]. Likewise, the stimulation with IL-8 increases the cell number and DNA synthesis of vascular smooth muscle cells [31]. Importantly, this is the first report to our knowledge demonstrating the *in vitro* effect of IL-17 on unstimulated neutrophils.

The lack of an upregulation by IL-17 of the pro-inflammatory cytokines we studied at 5 hours suggests that the effect of IL-17 on neutrophils is not immediate. These results are in agreement with the increased IL-17 induced neutrophil recruitment observed in mice after 24 hours, in comparison with 6 hours [32], and that the administration of neutralizing anti-IL-17 antibodies inhibits neutrophil recruitment at 24 hours, but not at 6 hours in mice [33].

### Viability and apoptosis

In our study, LPS significantly increased the apoptosis levels in equine neutrophils at 24 hours. Conversely, LPS at shorter culture times and at different doses than those used in our study is shown to cause a significant delay of human neutrophil apoptosis [34]. We hypothesized that this LPS-induced apoptosis rate could be influenced by incubation time and LPS doses. IL-17 significantly increased the viability of LPS-stimulated equine neutrophils by decreasing apoptosis levels after 24 hours in culture compared to the control. This finding is in agreement with the delayed apoptosis rate in IL-17-stimulated human neutrophils of tuberculosis-affected patients and healthy individuals [35]. This neutrophil response in human and horses appears to be species specific as conversely IL-17 rather induces neutrophil apoptosis in a mouse model of pneumococcal infection [36] as well as in peripheral blood murine neutrophils at 48h [37]. We also observed that IL-17 potentiated the dexamethasone-induced increase viability of equine neutrophils. Considering that IL-17 expression in peripheral blood from human patients with asthma and others diseases is unaltered by GCs [38, 39], IL-17 may hence contribute to persistent airway neutrophilia by promoting continuous neutrophil recruitment and increasing their viability. The IL-17-induced decrease in neutrophil apoptosis could influence the clearance of these cells from inflammatory sites and be detrimental to the resolution of inflammation.

### Receptors

We first examined the levels of mRNA and protein expression of IL-17RA and IL-17RC in highly purified equine neutrophils. This is because both subunits of the receptor are required in order for IL-17 to be biologically active. To our knowledge, this is the first report demonstrating the presence of both subunits of the IL-17 receptor at mRNA and protein levels on unstimulated peripheral blood neutrophils in any species. Previous studies reported the presence of IL-17RA in human neutrophils [5, 40], but IL-17RC was not observed in unstimulated or LPS-stimulated neutrophils [5]. However, IL-17RC was recently reported to be present in human neutrophils isolated from bone marrow after stimulation with one of two treatments: IL-6 and IL-23 alone or IL-6 and IL-23 in combination with *Aspergillus fumigatus* hyphal extracts. IL-17RC was not found in unstimulated cells [11]. The absence of IL-17RC in unstimulated neutrophils may be due to the short incubation time (only 3 hours) used in this study [11]. The sources of neutrophils (peripheral versus bone marrow neutrophils), method of isolation, and species differences could also contribute to the constitutive expression of IL-17RC we observed. Nevertheless, depending of the cell source, and microenvironment (presence of stimuli), human neutrophils can also express both subunits of the IL-17 receptor, making these cells reactive to IL-17. Further investigation should address the presence of these receptors in airway neutrophils of asthmatics and other lung diseases associated with an upregulation of IL-17.

## Conclusion

Previous studies have shown that IL-17 contributes to neutrophilic inflammation by inducing IL-8 mRNA expression by structural (smooth muscle cells, fibroblast, endothelial and epithelial cells) and inflammatory cells (macrophages), suggesting an indirect role of this cytokine in neutrophilic inflammation. Results of the present study indicate that IL-17 may also directly activate and possibly recruit neutrophils to the airways and that this response is not attenuated by DEX. Moreover, IL-17 increases the viability of blood neutrophils by decreasing apoptosis of these cells. We therefore speculate that the poor response to glucocorticosteroids observed in patients with severe asthma could be explained by the effect of IL-17 in the microenvironment of the lung. Further investigation is required to identify the signaling pathways involved in these biological activities. These results suggest that therapeutic strategies targeting or blocking IL-17 or its receptor subunits may be of benefit in the management of neutrophilic asthma.
